# European consensus on patient contact shielding

**DOI:** 10.1186/s13244-021-01085-4

**Published:** 2021-12-23

**Authors:** Peter Hiles, Patrick Gilligan, John Damilakis, Eric Briers, Cristian Candela-Juan, Dario Faj, Shane Foley, Guy Frija, Claudio Granata, Hugo de las Heras Gala, Ruben Pauwels, Marta Sans Merce, Georgios Simantirakis, Eliseo Vano

**Affiliations:** 1 Glan Clwyd Hospital, Bodelwyddan, Denbighshire UK; 2 European Federation of Organizations for Medical Physics, Utrecht, The Netherlands; 3 Mater Private Hospital, Eccles St., Dublin, Ireland; 4 European Society of Radiology - EuroSafe Imaging, Vienna, Austria; 5 University of Crete, Iraklion, Crete Greece; 6 Member ESR-Patient Advisory Group, Patient Advocate, Hasselt, Belgium; 7 Centro Nacional de Dosimetría (CND), Instituto Nacional de Gestión Sanitaria, Valencia, Spain; 8 European Radiation Dosimetry Group, Neuherberg, Germany; 9 Faculty of Dental Medicine and Health, Osijek, Croatia; 10 European Federation of Radiographer Societies, Utrecht, Belgium; 11 Radiography and Diagnostic Imaging, University College Dublin, Dublin, Ireland; 12 Université de Paris, Paris, France; 13 European Society of Paediatric Radiology, Le Kremlin-Bicêtre, France; 14 Institute for Maternal and Child Health, IRCCS “Burlo Garofolo”, Trieste, Italy; 15 Federal Office for Radiation Protection, Oberschleißheim, Germany; 16 Aarhus Institute of Advanced Studies, Aarhus University, Aarhus, Denmark; 17 Geneva University Hospitals, Geneva, Switzerland; 18 Greek Atomic Energy Commission, Agia Paraskevi, Athens, Greece; 19 Radiology Department, Complutense University, Madrid, Spain

**Keywords:** Consensus, Tomography (X-ray computed), Radiation dosage, Protective devices, Radiology

## Abstract

Patient contact shielding has been in use for many years in radiology departments in order to reduce the effects and risks of ionising radiation on certain organs. New technologies in projection imaging and CT scanning such as digital receptors and automatic exposure control systems have reduced doses and improved image consistency. These changes and a greater understanding of both the benefits and the risks from the use of shielding have led to a review of shielding use in radiology. A number of professional bodies have already issued guidance in this regard. This paper represents the current consensus view of the main bodies involved in radiation safety and imaging in Europe: European Federation of Organisations for Medical Physics, European Federation of Radiographer Societies, European Society of Radiology, European Society of Paediatric Radiology, EuroSafe Imaging, European Radiation Dosimetry Group (EURADOS), and European Academy of DentoMaxilloFacial Radiology (EADMFR). It is based on the expert recommendations of the Gonad and Patient Shielding (GAPS) Group formed with the purpose of developing consensus in this area. The recommendations are intended to be clear and easy to use. They are intended as guidance, and they are developed using a multidisciplinary team approach. It is recognised that regulations, custom and practice vary widely on the use of patient shielding in Europe and it is hoped that these recommendations will inform a change management program that will benefit patients and staff.

**Supplementary Information:**

The online version contains supplementary material available at 10.1186/s13244-021-01085-4.

## Key points

- Shielding use in radiology has been re-evaluated.
- Major European bodies involved in imaging radiation safety have issued consensus-based recommendations.
- This paper represents multidisciplinary based recommendations for shielding use.
- In the majority of cases patient contact shielding use is not recommended.

## Patient summary

Radiation used in radiology carries small risks of radiation damage. To minimise this damage to sensitive organs, contact shielding was used for many years. In contact shielding a shielding object (blanket, rubber mat…) with radiation absorbing material is used and placed in contact with the surface to be shielded. Recent technological advances in equipment and recent scientific knowledge, have led to new guidelines and they show that there is rarely a need for shielding, although it can sometimes be allowed. In some cases, shielding can even lessen the quality of imaging or increase radiation dose. However, in case the patient has any doubts this should be discussed with the radiographer or other imaging professional.

## Introduction

In the healthcare sector, radiation protection devices are frequently placed in contact with the human body to reduce the radiation exposure to radiosensitive organs of patients undergoing diagnostic and interventional X-ray examinations. Such patient contact shielding has been in widespread use for the last seventy years, aiming to protect against genetic effects, cancer and other detriment [1].

However, an increasing number of studies, position statements and recommendations have raised concerns regarding the utility and effectiveness of such shielding [2–5]. This has added to an unhelpful and undesirable inconsistency in regulation and recommendations of shielding use across Europe [6].

The growing need for a European consensus statement on patient contact shielding has been highlighted by Gilligan and Damilakis [7], with the main objective of supporting and promoting effective and harmonised clinical practice.

Representatives of the European Federation of Medical Physicists (EFOMP), European Federation of Radiographer Societies (EFRS), European Society of Radiology (ESR), European Society of Paediatric Radiology (ESPR), EuroSafe Imaging (ESI), European Radiation Dosimetry Group (EURADOS) and European Academy of DentoMaxilloFacial Radiology (EADMFR), as well as a representative from the Patient Advisory Group of ESR, founded the GAPS (Gonad and Patient Shielding) group (chair: P Gilligan) with the purpose to propose a European recommendation on the use of contact shielding.

## Evidence review criteria

This consensus statement has involved examining the evidence-base provided in published data and guidance. The system of ranking the evidence is based on a user-friendly system developed by the European Heart Rhythm Association, EHRA [8] and here uses ‘coloured shields’ that provide an indication of the current status of the evidence and consequent guidance (see Table 1).

**Table 1:** Rationale for consensus statements

Rationale	Consensus Recommendation	Symbol
Evidence that using patient contact shielding is beneficial and effective	‘Should use shielding’	
General agreement favours usefulness of patient contact shielding in some circumstances	‘May use shielding’	
Evidence or general agreement not to use patient contact shielding	‘Not recommended to use shielding’	

Thus, a green shield indicates a ‘should do this’ consensus statement or indicated risk assessment strategy based on strong evidence that it is beneficial and effective. An amber shield indicates general agreement and/or scientific evidence favouring a ‘may do this’ statement or the usefulness/efficacy of a risk assessment strategy or procedure. Risk assessment strategies for which there is scientific evidence of little or no benefit or even potential harm and should not be used (‘Not recommended to do this’) are indicated by a red-striped shield.

## Guidelines for clinical practice

Research has previously reported dose reductions of 30–95% to individual organs using shielding [9–11]. However, there has been a growing body of evidence that patient contact shielding is ineffective in most situations and at times potentially hazardous. The use of contact shielding can provide false reassurance (to patients and staff) and continued use can overemphasise the hazards of ionising radiation in the public mind.

This has led to an inconsistency of application of shielding. In some cases it has also led to conflict between patient expectations that shielding would be used and professionals judging it as unnecessary or even harmful.

The main aim of this consensus statement is to encourage and support good clinical practice by promoting harmonisation of application of patient contact shielding. This statement should be seen as a tool for making decisions in healthcare more rational and for improving the quality of healthcare delivery. However, it should not serve as a substitute for sound clinical judgement nor replace professional responsibility of providers.

This consensus statement is intended to help in the development of local policies and procedures by highlighting the reported limited utility and potential drawbacks of patient contact shielding.

Section “Issues when using contact shielding” also considers scenarios and approaches where individual circumstances such as high cumulative dose, anxious or radiosensitive patients may indicate that the radiology professional chooses to use shielding.

## Evidence for change

### Decrease in patient doses

While the number of X-ray imaging examinations has increased during the last decades, individual patient doses have decreased significantly since patient contact shielding was first introduced [12], limiting the potential benefit of this shielding in most cases. Although some patients may be exposed to high cumulative radiation doses due to multiple examinations [13], or in complex interventional procedures [14], the highest doses are absorbed by organs and tissues being imaged, which cannot generally be shielded (see Section “Shielding within the imaging field of view (FOV)”). Therefore, currently, only a minor number of patients might experience a real benefit from using contact shielding, which also comes with a risk, as discussed next.

Past practice in radiation protection has been based on the dose range and associated risk estimates prevalent at the time. However, the levels of dose and the organs- and age-risks estimates have changed over the years (see Section “Patient radiation risk from imaging”), requiring continuous revision of local practice in line with current knowledge and advice [4].

### Shielding within the imaging field of view (FOV)

There are several factors to be considered when applying patient contact shielding within the imaging FOV. These include:

- Incorrect placement of shielding by the operator or unintended movement of the shield during the examination can obscure important pathologies in the image, requiring repeat exposure [15].
- The operator may encounter difficulties in correct placement of shielding to cover intended radiosensitive organs due to variation in patients’ anatomy [16]. This may only be apparent after the image has been taken and can give rise to ineffective shielding.
- The highly attenuating material of the shielding may interfere with automatic exposure control systems and can lead to an increase rather than a decrease in patient dose [3, 17].
- Beam hardening or streak artefacts caused by the applied shielding can reduce the image quality and may lead to the requirement to repeat the exposure [18].

### Shielding outside the imaging FOV

The majority of scatter is internal and therefore cannot be shielded externally. Scatter doses are considerably smaller than the dose to anatomy within the area of interest or imaging FOV. As the patient doses have decreased over the years so too has the dose due to scattered radiation, which has now reduced to negligible levels in many cases. The probable benefits from the very small dose reduction due to contact shielding may not outweigh the potential risks of artefacts, infection and patient discomfort, as referenced below.

The placing of out of beam protection beyond the irradiated volume is not necessarily a simple, error free, task. For example, in helical CT scanning, there is a requirement to ‘overscan’ beyond the first and last image position in order to provide enough data to interpolate for those images. Since even a small amount of ‘overscan’ can extend a considerable distance beyond the image volume, placing a patient contact shielding adjacent to the scan volume can interfere with the image reconstruction leading to artefacts in the image [4].

### Patient radiation risk from imaging

The primary concern when justifying a medical exposure is the risk–benefit balance. Therefore, the approach to deciding on adopting or avoiding patient contact shielding should centre on the change in radiation dose and risk. For example, in some cases the application of contact shielding is reported to show a large relative dose reduction to a specific organ, giving the impression of a significant improvement, whereas the absolute benefit may be small or non-existent [2].

In addition, the focus of patient radiation safety should be upon those organs deemed to be at risk from cancer induction due to radiation exposure.

**Fig. 1:**
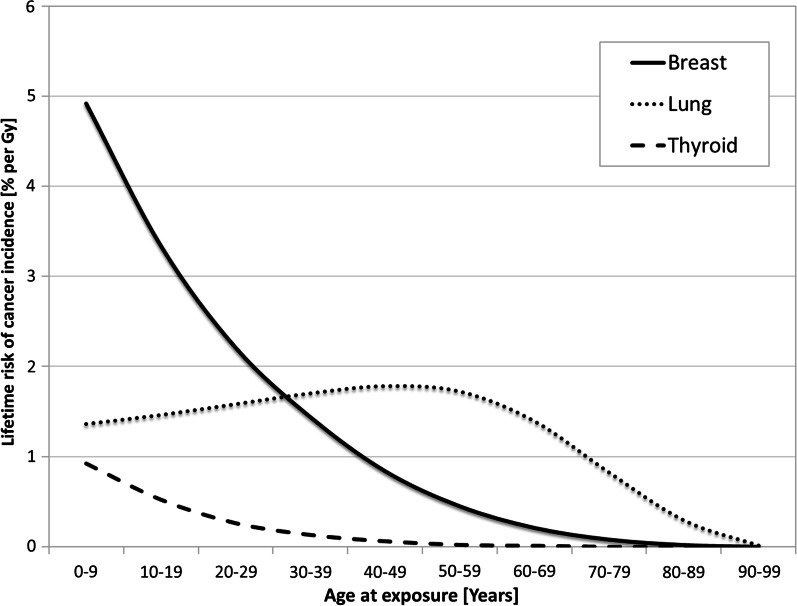
Female lifetime risks of cancer incidence by organ and age for a composite Euro-American population (% per Gy), based on data from reference [19]

However, when reviewing the need to protect a specific organ, it is important to take into account the fact that the radiation risk actually varies with age and sex of the patient, as illustrated in Fig. 1. This highlights the fact that paediatric patients can be at high risk and that the organ at highest risk can change with age.

## Recommendations

These recommendations are divided into areas of the body where patient shielding may be used and assume that all other applicable justification and optimisation strategies have been employed before patient contact shielding is considered.

For example, in general radiography, with good collimation and using posterior anterior (PA) positioning for skull, spinal and chest X-rays, patient contact shielding is likely to have a negligible benefit and, in many instances, may obscure diagnostic information or lead to an overall increase in patient dose. A summary of the recommendations in this consensus document is provided as an appendix (Additional file 1).

### Gonad shielding

Protection of the gonads is the longest-standing use of patient contact shielding due to the perception of the risk and the relative ease of use. However, genetic effects from radiation have not been observed in human studies despite the public perception otherwise. Indeed ICRP 103 [21] reduced the tissue weighting factor for the gonads to less than half its previous figure (0.2 to 0.08). Therefore, gonad shielding is perhaps the least useful in terms of reducing the radiation risk for the patient. Hereditable effects associated with typical dose ranges are likely to be negligible.

Within the FOV, there is a general consensus that it is difficult to position the shielding for female patients to ensure coverage of the ovaries, as well as avoiding interference with the anatomy of interest and the automatic exposure control system. Current published evidence has shown inconsistent results and disappointing impact on accuracy of shield application following audit and training’ [16].

Outside the FOV the reduction in radiation risk for both male and female patients by using shielding is negligible, regardless of age [2].

For CT scanning of the abdomen, several papers have shown a range of measured testicular dose reductions (58% to 95%) through the use of outside field of view wraparound and testicular shields in male adults and phantoms [10, 20]. In terms of absolute risk reduction based on a LNT model (given the limitations of uncertainty), this is of the order of 0.5 in 10,000 [22]. The benefit is small compared to other optimisation techniques such as limiting scan range in the area of the more radiosensitive organs as defined by the ICRP [21], and also comes with some risks. Yu et al. [23] showed that such shields provided little benefit in paediatric chest CT too as one got further from the field of view. There are risks of interfering with the automatic exposure control when using shielding outside the field of view such as those found in embryo shielding [24].

**Table:** 

Application	Imaging modality	Inside or outside FOV	Recommendation	Symbol
Male and female gonad contact shielding	All X-ray	Both	‘Not recommended to use shielding’	

### Thyroid shielding

The thyroid gland has been highlighted as a radiosensitive organ. Since the relative sensitivity of the thyroid gland to radiation-induced cancer is strongly biased towards children and there is a longer time for any induced cancer to manifest itself, it is particularly important to consider this age group when deciding if thyroid protection is required, particularly when high cumulative radiation doses are expected due, for example, to multiple head CT examinations.

Since the shield should cover the front half of the neck, it can readily interfere with the imaging process within the FOV (see Section “Shielding within the imaging field of view (FOV)”). Outside the FOV, the effectiveness in reducing patient stochastic risk is minimal.

Whilst it is generally considered that patient contact shielding should not be used, exceptions may exist in the field of dental X-ray imaging due to the proximity of the thyroid to the FOV and the high percentage of paediatric patients examined [25–27].

In cephalometric radiography, a conventional thyroid collar can partially overlap with the FOV. However, thyroid shielding can be applied, if evaluation of the cervical spine is not needed [28, 29] or custom protective devices that do not overlap with relevant anatomical regions are used [30].

If shielding were to be used, it is strongly recommended that a Medical Physics Expert (MPE) is consulted first, as there is the potential to introduce artefacts to the image should a thyroid collar enter the useful imaging volume. In addition, increased patient doses can arise from systems (e.g. CBCT) that incorporate an automatic exposure system [27].

**Table:** 

Application	Imaging modality	Inside or outside FOV	Recommendation	Symbol
Thyroid contact shielding	All X-ray (except Ceph.)	Inside	‘Not recommended to use shielding’	
Thyroid contact shielding	Cephalometric radiography	Inside	‘May use shielding’	
Thyroid contact shielding	Radiography, Mammography, Fluoroscopy, CT	Outside	‘Not recommended to use shielding’	
Thyroid contact shielding	Dental intraoral and cephalometric radiography	Outside	‘May use shielding’	
Thyroid contact shielding	CBCT	Outside	‘May use shielding’	

### Breast shielding

In a similar manner to the thyroid gland, breast tissue is highly sensitive to radiation, particularly for those less than 30 years of age.

Since the shield should cover the anterior surface of the chest, if it is within the FOV it could compromise the X-ray examination and give rise to an increased radiation dose to neighbouring organs and tissues. For example, in CT chest examinations of patients over 30 years old, the lung is the most radiosensitive organ (see Section “Patient radiation risk from imaging”) and using breast contact shielding could lead to an increased lung dose, thus increasing, rather than decreasing, the overall risk to the patient.

Outside the FOV, the effectiveness in reducing patient stochastic risk is generally reported to be minimal [2].

**Table:** 

Application	Imaging modality	Inside or outside FOV	Recommendation	Symbol
Breast contact shielding	All X-ray	Both	‘Not recommended to use shielding’	

### Eye lens shielding

The lens of the eye is considered one of the most radiosensitive tissues in the body, with the primary concern being the development of cataracts and lens opacities following radiation exposure. However, in the case of CT, most recent studies suggest that dose reduction strategies are more effective than eye shielding (e.g., [31]). Due to the level of eye dose for some fluoroscopically guided cerebral interventional procedures [32, 33], the consultation of a Medical Physics Expert is advised on a case-by-case basis.

**Table:** 

Application	Imaging modality	Inside or outside FOV	Recommendation	Symbol
Eye lens contact shielding	All X-ray	Both	‘Not recommended to use shielding’	

### Embryo/Fetal shielding

Studies have shown that radiation protection shields have limited value for the protection of the unborn child from examinations performed on pregnant patients because most of the embryo/fetal exposure results from internal scatter in the tissues of the mother [34, 35]. In addition, if suitable optimisation strategies are adopted, the impact of patient contact shielding on the fetal dose is minimal [36].

Any discussion around this may require sensitive handling. Pregnant patients undergoing diagnostic radiology examinations may request abdominal protection, including situations where the examination is outside the pelvic region. In these cases, whether or not to provide extra shielding, usually in the form of lead/lead-equivalent material draped over the abdomen, should be in accordance with written procedures and at the discretion of the operator performing the imaging. If a decision is made to use contact shielding, then it is important that accurate collimation is used, and the shielding must not encroach on the automatic exposure control system. This includes taking account of any ‘overscan’ (see Section “Shielding outside the imaging FOV”) beyond the first and last image position.

**Table:** 

Application	Imaging modality	Inside or outside FOV	Recommendation	Symbol
Embryo / Fetal contact shielding	All X-ray	Inside	‘Not recommended to use shielding’	
Embryo / Fetal contact shielding	Radiography, Mammography, Fluoroscopy, Dental Radiography, CT	Outside	‘Not recommended to use shielding’	

## Issues when using contact shielding

It is not unreasonable to consider scenarios and approaches where individual circumstances such as high cumulative dose, anxious or radiosensitive patients may indicate to the radiology professional that the benefit of shielding could outweigh any risk associated with its use. While not generally advised, any use of contact shielding should be considered carefully by a multi-disciplinary team, with the advice of a MPE, and should be written into examination protocols ahead of use.

Its selection simply to reassure the apprehensive patient should be discouraged as this promotes mixed messages and an exaggeration of radiation risk to the patient and wider community. Instead, efforts should concentrate on explaining the risks from the use of contact shields to the patient [4].

Besides the risks of artefacts and interference with the AEC system, a disadvantage to using shielding is the potential discomfort experienced by the patient and the manual handling issues for the staff [9], as well as potential infection control issues [37, 38]. Furthermore, the use of shielding may not be advisable for emergency patients, paediatrics or individuals with disabilities who are unable to tolerate the use of the shield (e.g., eye lens shielding).

Where it is agreed that shielding should be used, then staff should be trained in:

- The selection of appropriate shielding, including how to prevent shielding moving during a procedure due to patient or equipment movement (e.g., during dynamic imaging)
- The selection of appropriate radiographic techniques, including how to avoid interference with automatic exposure control systems
- How to perform quality control checks on patient contact shielding
- How to store shielding appropriately
- How to clean and disinfect shielding
- How to comply with local policies regarding patient dignity (e.g., transgender patients [39])
- Communication skills specific to discussions with patients, parents or caretakers of children undergoing radiological examinations and healthcare professionals on the use of patient contact shielding.
- How to communicate benefit risk to pregnant patients

## Next steps

For some users of radiation, the implementation of this guidance and recommendations may represent a significant cultural change in practice and require development of a change management program, with stakeholder consultation.

Following the adoption of this consensus statement, there will be a need to review current practice and provide suitable information and education material for health professionals and the public.

The European Society of Radiology through Eurosafe Imaging, with the assistance of the GAPS group (see introduction), are currently planning the first step, through a web-based survey of Radiology departments to evaluate the current practice of contact shielding within Europe.

A concerted effort will be required by the relevant professional bodies to ensure the next steps of education and training to explain the changes in guidance are made readily available to European users. Some useful information on patient shielding is already available online, including the British Institute of Radiology (https://www.bir.org.uk/education-and-events/patient-shielding-guidance.aspx) and the American Association of Physicists in Medicine CARES (Communicating Advances in Radiation Education for Shielding) group (https://w3.aapm.org/cares/).

## Review of current guidelines

The technology used in X-ray imaging, the level of radiation doses absorbed by the patients and the knowledge on radiation dose effects due to ionising radiation may vary over time. Therefore, it is deemed necessary to review these guidelines periodically. In principle, these will be reviewed after a period of five years or sooner if new evidence or changes recommend so.

## Supplementary Information

**Additional file 1. Appendix 1** Summary of recommendations

## Data Availability

All relevant data is included in this publication.

## Abbreviations

CT: Computed tomography
FOV: Field of view
MPE: Medical physics expert
