# Supplementary material for: European consensus on patient contact shielding
Source: Insights Imaging. 2021 Dec 23;12:194. doi: 10.1186/s13244-021-01085-4 (PMC8695402; doi:10.1186/s13244-021-01085-4)
Supplement: Supplementary file 1 — Additional file 1. Appendix 1 Summary of recommendations [file 13244_2021_1085_MOESM1_ESM.docx]

# ELECTRONIC SUPPLEMENTARY MATERIAL

# Appendix 1 Summary of recommendations

See main text for detail on recommendations.

| Application | Imaging modality | Inside or outside FOV | Recommendation | Symbol |
| --- | --- | --- | --- | --- |
| Male and female gonad contact shielding | All X-ray | Both | ‘Not recommended to use shielding’ | 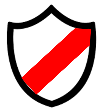 |
| Thyroid contact shielding | All X-ray (except Ceph.) | Inside | ‘Not recommended to use shielding’ | 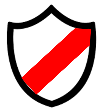 |
|  | Cephalometric radiography | Inside | ‘May use shielding’ | 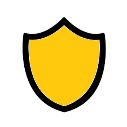 |
|  | Radiography, Mammography, Fluoroscopy, CT | Outside | ‘Not recommended to use shielding’ | 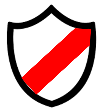 |
|  | Dental X-ray (intraoral and cephalometric) | Outside | ‘May use shielding’ | 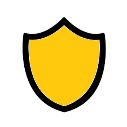 |
|  | CBCT | Outside | ‘May use shielding’ | 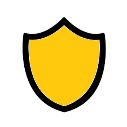 |
| Breast contact shielding | All X-ray | Both | ‘Not recommended to use shielding’ | 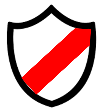 |
| Eye lens contact shielding | All X-ray | Both | ‘Not recommended to use shielding’ | 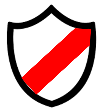 |
| Embryo / Fetal contact shielding | All X-ray | Both | ‘Not recommended to use shielding’ | 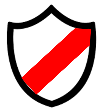 |
